# Complete chloroplast genome of the plant *Stahlianthus Involucratus* (Zingiberaceae)

**DOI:** 10.1080/23802359.2019.1644227

**Published:** 2019-07-23

**Authors:** Dong-Mei Li, Ye-Chun Xu, Gen-Fa Zhu

**Affiliations:** Guangdong Key Lab of Ornamental Plant Germplasm Innovation and Utilization, Environmental Horticulture Research Institute, Guangdong Academy of Agricultural Sciences, Guangzhou, China

**Keywords:** *Stahlianthus involucratus*, Zingiberaceae, chloroplast genome, phylogenetic analysis, single nucleotide polymorphism

## Abstract

The first complete chloroplast genome of *Stahlianthus involucratus* (Zingiberaceae) was reported in this study. The *S. involucratus* chloroplast genome was 163,300 bp in length and consisted of one large single copy (LSC) region of 87,498 bp, one small single copy (SSC) region of 15,568 bp, and a pair of inverted repeat (IR) regions 30,117 bp. It encoded 141 genes, including 87 protein-coding genes (79 PCG species), 46 tRNA genes (28 tRNA species) and 8 rRNA genes (4 rRNA species). The phylogenetic analysis based on single nucleotide polymorphisms strongly supported that *S. involucratus, Curcuma roscoeana* and *Curcuma longa* formed a cluster in group *Curcuma*II within family Zingiberaceae.

*Stahlianthus involucratus* (King ex Baker), also known as ‘Tu Tian Qi’ in Chinese, is a species of perennial herb in genus *Stahlianthus* family Zingiberaceae (Wu and Larsen 2000; Wu et al. 2016). *S. involucratus* naturally occurs at forest floors and mountain slopes (Wu and Larsen 2000). It is mainly distributed in the regions of Southern and Northwestern China (Guangdong, Guangxi, Fujian, Hainan and Yunnan provinces), India, Myanmar, and Thailand (Wu and Larsen 2000; Wu et al. 2016). Morphological classification of *Stahlianthus* species was difficult owing to the morphological similarity of vegetative parts among species and the genus *Kaempferia* in Zingiberaceae (Wu and Larsen 2000). For instance, in morphological classification, *S. involucratus*, originally named as *Kaempferia involucratus* in 1890 by King ex Baker in J.D. Hooker, this very well-known species was transferred to *Stahlianthus* by Craib ex Loesener in 1930 (Wu and Larsen 2000). Therefore, based only on morphological characteristics, we could not conclusively distinguish and identify the *Stahlianthus* species and the genus *Kaempferia* species in family Zingiberaceae. Within family Zingiberaceae, reports on complete chloroplast genome sequences are still very scarce (Wu et al. 2017; Li, Zhao, et al. 2019), hindering phylogenetic analyses based on large scale chloroplast genomes. Nevertheless, no complete chloroplast genome belonging to genus *Stahlianthus* has been reported.

*Stahlianthus Involucratus* was collected from Jinghong, Yunnan province and stored at the resource garden of Environmental Horticulture Research Institute (specimen accession number Si2015), Guangdong academy of agricultural sciences, Guangzhou, China. Total chloroplast DNA was extracted from about 100 g of fresh leaves of *S. involucratus* using the sucrose gradient centrifugation method (Li et al. 2012). Chloroplast DNA (accession number SiDNA2017) was stored at −80 °C in Guangdong key lab of ornamental plant germplasm innovation and utilization, Environmental Horticulture Research Institute, Guangdong Academy of Agricultural Sciences, Guangzhou, China. Library construction was using Illumina (Illumina, CA, USA) and PacBio (Novogene, Beijing, China) sequencing, respectively. The Illumina and PacBio sequencing data were deposited in the NCBI sequence read archive under accession numbers SRR8189638 and SRR8184505, respectively. After trimming, the Illumina sequencing and PacBio sequencing yielded 66.3 M clean data of 150 bp paired-end reads and 0.89 M clean data of 8–10 kb subreads, respectively. The chloroplast genome of *S. involucratus* was assembled and annotated by using the reported methods (Li, Wu, et al. 2019). The complete chloroplast genome sequence of *S. involucratus* was submitted to GenBank (accession number: MK262725).

The complete chloroplast genome of *S. involucratus* was 163,300 bp in length and comprised a pair of inverted repeat (IR) regions of 30,117 bp each, a large single-copy (LSC) region of 87,498 bp, and a small single-copy (SSC) region of 15,568 bp. It was predicted to contain a total of 141 genes, including 46 tRNAs (28 tRNAs species), 87 protein-coding genes (79 PCG species) and 8 rRNAs (4 rRNAs species). Twenty species genes occurred in double copies, including 8 PCG genes (*ndhB*, *rpl2*, *rpl23*, *rps7*, *rps12*, *rps19*, *ycf1*, *ycf2*), 8 tRNAs (*trnH-GUG*, *trnI-CAU*, *trnL-CAA*, *trnV-GAC*, *trnI-GAU*, *trnA-UGC*, *trnR-ACG*, *trnN-GUU*) and 4 rRNAs (*rrn4.5*, *rrn5*, *rrn16* and *rrn23*). All these 20 species genes were located in the IR regions. Most of the PCG genes contained only one exon, while 17 genes contained one or two introns. Out of the 17 intron-containing genes, 10 PCG genes (*atpF*, *ndhA*, *ndhB*, *rpoC1*, *petB*, *petD*, *rpl2*, *rpl16*, *rps12* and *rps16*) and 5 tRNAs (*trnK-UUU*, *trnL-UAA*, *trnV-UAC*, *trnI-GAU* and *trnA-UAC*) had a single intron, while two other genes (*ycf3* and *clpP*) possessed two introns. The nucleotide composition was asymmetric (31.73% A, 18.32% C, 17.68% G, 32.27% T) with an overall AT content of 64.00%. The AT contents of the LSC, SSC, and IR regions were 66.22%, 70.41%, and 59.11%, respectively.

To obtain its phylogenetic position within family Zingiberaceae, a molecular phylogenetic tree was constructed by using single nucleotide polymorphisms (SNPs) arrays from available 14 species chloroplast genomes using *Costus viridis, Costus pulverulentus,* and *Canna indica* as outgroup taxa. The SNP arrays were obtained as previously described method (Li, Zhao, et al. 2019). For each chloroplast genome, all SNPs were connected in the same order to obtain a sequence in FASTA format. Multiple FASTA format sequences alignments were carried out using ClustalX version 1.81 (Thompson et al. 1997). A maximum likelihood phylogenetic tree (Figure 1) was constructed using the SNPs from 14 chloroplast genomes alignment result with MEGA7 (Kumar et al. 2016). As shown in the phylogenetic tree (Figure 1), *S. involucratus, Curcuma roscoeana,* and *Curcuma longa* formed a cluster in group *Curcuma*II within family Zingiberaceae.

**Figure 1.**
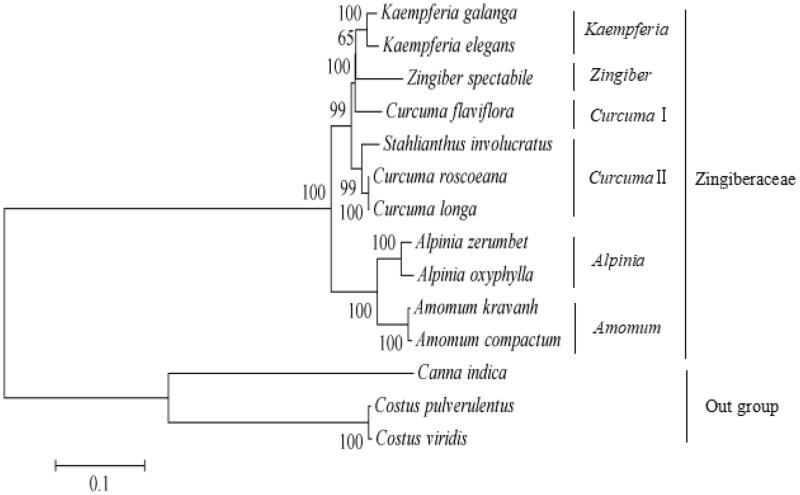
Phylogenetic tree constructed with single nucleotide polymorphisms arrays from 14 species chloroplast genomes using maximum likelihood method. The bootstrap values were based on 1,000 replicates and are indicated next to the branches. Accession numbers: *Alpinia zerumbet* JX088668, *Alpinia oxyphylla* NC_035895.1, *Curcuma flaviflora* KR967361, *Zingiber spectabile* JX088661, *Curcuma roscoeana* NC_022928.1, *Curcuma longa* MK262732, *Kaempferia galanga* MK209001, *Kaempferia elegans* MK209002, *Amomum kravanh* NC_036935.1, *Amomum compactum* NC_036992.1, *Costus pulverulentus* KF601573, *Costus viridis* MK262733 and *Canna indica* KF601570.
